# Clinical Implementation and Oncological Relevance of Molecular Profiling in Brain Metastases Patients—A Multicenter Retrospective Cohort Study

**DOI:** 10.1002/ijc.70611

**Published:** 2026-06-18

**Authors:** Maria Nikolaeva, Jacopo Bellomo, Meltem Gönel, Vittorio Stumpo, Victor Egon Staartjes, Flavio Vasella, Kevin Akeret, André Sagerer, Anna Maria Zeitlberger, David Wasilewski, Christopher Krämer, Sven König, Julia Onken, Tareq Juratli, Alexander Köpp, Regina Reimann, Marcus Czabanka, Peter Vajkoczy, Ilker Y. Eyüpoglu, Oliver Bozinov, Michael Weller, Emilie Le Rhun, Luca Regli, Carlo Serra, Marian C. Neidert, Stefanos Voglis

**Affiliations:** ^1^ Department of Neurosurgery University Hospital and University of Zurich Zurich Switzerland; ^2^ Department of Neurosurgery Medical Faculty and University Hospital Carl Gustav Carus, TUD Dresden University of Technology Dresden Germany; ^3^ Division of Neurosurgery ΗOCH, Cantonal Hospital St. Gallen St. Gallen Switzerland; ^4^ Department of Neurosurgery German Cancer Consortium (DKTK) Partner Site Berlin Charité – University Hospital Berlin Berlin Germany; ^5^ Department of Neurosurgery University Hospital Düsseldorf Düsseldorf Germany; ^6^ Department of Neurosurgery University Hospital Frankfurt Frankfurt Germany; ^7^ Department of Neurology University Hospital and University of Zurich Zurich Switzerland; ^8^ Institute of Neuropathology, University Hospital and University of Zurich Zurich Switzerland; ^9^ Department of Medical Oncology and Hematology University Hospital and University of Zurich Zurich Switzerland

**Keywords:** driver mutations, molecular profiling, precision oncology, targeted therapy

## Abstract

Brain metastases (BM) require a multidisciplinary treatment approach combining surgery, radiotherapy, and systemic therapy. While current guidelines recommend the analysis of established cancer driver genes in BM tissue, little is known about its real‐life implementation and relevance on oncological treatment strategies. This retrospective multicenter study includes patients with BM from melanoma, lung and breast cancer operated between 2010 and 2022. Clinical records were evaluated for BM molecular analyses of predefined cancer drivers and their discordance to extracranial tumor sites, that is, ALK, BRAF, EGFR, KRAS, NTRK for lung, HER2, estrogen/progesterone receptor, BRCA1/2 for breast cancer and BRAF, KIT, NRAS among others for melanoma. Adjustment of systemic therapies based on BM molecular profiles was analyzed. Among 1431 BMs screened for availability of molecular analysis, molecular profiling was performed at least partially in 723 BMs (51%). In cases with matched extracranial tumor samples (*n* = 276), discordant alterations were found in 18% of lung, 4.7% of melanoma and 45% of breast cancer BMs. Molecular BM analyses informed systemic therapy adjustments in 13%–27% of patients depending on the primary tumor. Overall survival was significantly better in patients who underwent BM profiling, especially when operated in recensst years (median 19.3 vs. 9.9 months; *p* < 0.0001) and in patients with breast cancer who had a change in systemic therapies upon BM profiling (*p* = 0.0085). In conclusion, molecular profiling of BM allows selecting available treatment options for a substantial subset of patients. This study underscores the need for more systematic molecular testing in BM patients to guide systemic treatment decisions.

## What's New?

While current guidelines recommend the analysis of established cancer driver genes in brain metastases, little is known about its real‐life implementation. This multicenter study revealed an upward trend in the profiling rates of surgically treated brain metastases over the past decade, with up to 60% of brain metastases samples undergoing analysis. The molecular analyses informed systemic therapy adjustments in 13–27% of patients, depending on the primary tumor. Overall survival was significantly better in patients who received brain metastases profiling. This study underscores the need for more systematic molecular testing in patients with brain metastases to guide systemic treatment decisions.

## Graphical abstract

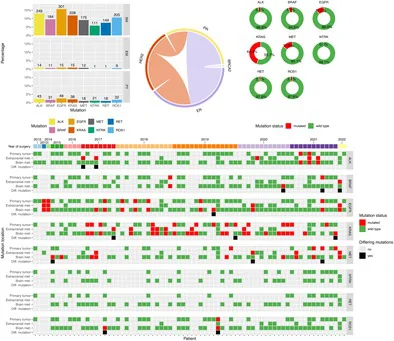

**Abbreviations**
BM: brain metastases
CNS: central nervous system
EM: extracranial metastases
ER: estrogen receptor
IHC: immunohistochemical
IRS: immunoreactive score
NSCLC: non‐small cell lung cancer
OS: overall survival
PR: progesterone receptor
PT: primary tumor

## 1 Introduction

Brain metastases (BM) are the most common malignant intracranial tumors that affect around 10%–20% of all cancer patients [1, 2], have an increasing incidence due to improved treatment options for many solid cancer types [3] and remain prognosis limiting with a high morbidity and mortality in these patients [4].

While surgery [5, 6, 7, 8, 9], radiotherapy and conventional chemotherapy are historically the mainstay in BM treatment, emerging molecular therapies have led to significantly increased survival rates, notably in patients with actionable genetic alterations [10, 11, 12, 13]. A substantial subset of tumors—up to 57% in one study [14]—harbor clinically actionable driver mutations. However, the frequency of these mutations in BM tissue remains largely unknown. Nevertheless, targeted therapeutic interventions in BM tissue should be informed by the molecular characterization of BM tissue, as studies have shown that the molecular profile of BM is not necessarily recapitulated in matched primary tumors (PTs) or extracranial metastatic sites [15, 16]. This is due to the occurrence of early branched clonal evolution and selective pressures that drive treatment resistance within the BM niche. BM molecular profiling revealed discordance rates of up to 67% for EGFR status in non‐small cell lung cancer (NSCLC) [17], 14% for BRAF in melanoma [18], and 52% for receptor conversion in breast cancer [19], requiring not only frequent therapy adjustments but also providing valuable information for disease prognostication. The determination of the molecular status represents a determinant source of information to guide the systemic therapeutic decisions.

Importantly, targeted and immunotherapeutic treatments have been shown to prolong survival in some patients with BM across several PT types, although they have lower response rates compared to extracranial tumor manifestations [10, 11, 12, 13]. These findings underscore the role of the central nervous system (CNS) as a unique metastatic niche, prompting the need for distinct diagnostic and therapeutic considerations. In BM from breast and lung cancer, standard treatment is guided by molecular subtype, with targeted agents forming a first‐line component of multimodal management in oncogene‐driven NSCLC and HER2‐positive breast cancer [20]. In melanoma BM, immune checkpoint inhibitors are currently recommended as first‐line treatment regardless of BRAF mutation status [21], given their durable intracranial disease control and improved long‐term survival outcomes [11, 22]. Targeted therapies nevertheless remain an important option in selected situations, such as symptomatic metastases or contraindications to immunotherapy, owing to their rapid antitumor effects [21, 23] and are being investigated in combination approaches [24, 25].

Accordingly, present guidelines recommend the analysis of entity‐specific molecular markers in all resected BM [20, 26, 27]. While broader testing availability and increased awareness render this approach theoretically feasible [28], its practical implementation and clinical utility in the management of BM patients remain insufficiently understood.

In this study, we investigate the application of molecular profiling in BM from lung cancer, melanoma, and breast cancer across five tertiary neurosurgical centers. Specifically, we assess the frequency of targetable molecular alterations, their discordance with matched extracranial tumor samples, and the resulting implications for oncological treatment strategies.

## 2 Methods

### 2.1 Study Cohort and Data Acquisition

Patient data were retrospectively retrieved from institutional BM patient registries for all surgically resected or biopsied lung cancer, breast cancer, or melanoma BM and screened for availability of molecular profiling. Inclusion criteria included histologically confirmed BM from these PT types, absence of opposition to further use of clinical data, and the inclusion timeframe with BM surgeries between 2010 and 2022, although some institutional registries lacked complete datasets at some years.

### 2.2 Assessment of Molecular Profiling and Change in Systemic Therapy

If molecular profiling of BM was available, clinical records were further evaluated for diagnostic information on PTs and extracranial metastases (EM). Treatment‐relevant molecular alterations were defined according to current EANO‐ESMO guidelines [26]. Overall, this included HER2, estrogen/progesterone receptor (ER/PR), BRCA1/2, PIK3CA in breast cancer; ALK, BRAF, EGFR, KRAS, NTRK, MET, RET, ROS1 in lung cancer and BRAF, KIT, NF1, NRAS in melanoma.

Marker status was recorded in an assay‐agnostic manner, as routine diagnostic modalities differed between centers and changed over time and were not the focus of our research question. For melanoma and lung cancer BM, molecular profiling was limited to results from genetic analyses using tumor‐derived gDNA, including single‐gene sequencing or PCR‐based assays, panel‐based next‐generation sequencing (NGS), and fluorescence in situ hybridization (FISH). In breast cancer, molecular profiling comprised genetic testing as well as immunohistochemical (IHC) assessment of hormone receptor status. The specific assays are detailed in Table S1. Additionally, detected molecular changes in the BM were summarized based on their exact mutation/protein changes—as recorded by the participating study centers—in Table S2. IHC receptor status was interpreted by respective pathologists, for breast cancer samples commonly defined as positive with ER/PR ≥ 1% positivity immunoreactive score (IRS) 1 [29] and HER2 score of minimum 3+ or 2+ with positive Her2‐FISH [30]. One center only retrieved data from molecular genetic testing.

Based on the available therapeutic agents in the studied time period, drug classes were broadly categorized according to the molecular pathways they are targeting [31], that is, tyrosine kinase inhibitors against oncogenic fusion genes (ALK, NTRK, ROS1, RET, MET) or EGFR mutations, KRAS inhibitors, BRAF/MEK inhibitors, HER2‐specific therapies, immune modulating therapies, and others (including PARP‐, CDK‐, KIT‐targeted therapies or study inclusion). We evaluated the BM‐specific change of targeted treatment, defined as start or discontinuation of any systemic drug class based on BM molecular profiling results as documented in clinical records and tumor board reports.

### 2.3 Statistical Analysis

All data processing and statistical analyses were performed using R (version 4.4.1) [32] and R Studio (version 2024.12.1, Posit Software) [33] with open‐source libraries. Patient and lesional characteristics are presented as percentages for categorical variables. Missing values were considered as missing at random and therefore omitted from the analyses. *p* values < 0.05 were considered statistically significant. As the statistical analysis was exploratory, no correction for multiple testing was applied. Overall survival (OS) was calculated from the time of surgery, and survival analysis was performed using the log‐rank statistic. To address the potential confounding factor of timing of surgery, given that the availability of various targeted therapies, and also the molecular profiling of BM, increased over time, the cohort was divided into two groups based on the year of surgery: patients operated on between 2011 and 2016 versus those operated on between 2017 and 2022.

## 3 Results

### 3.1 Study Cohort and BM Characteristics

A total of 1431 patients with BM from five participating neurosurgical centers were screened for molecular profiling (Table 1). The majority of surgeries (*n* = 1328, 93%) were first‐time resections of BM, and in 82% (*n* = 747) the BM diagnosis was triggered by neurological symptoms and signs. This was followed by diagnoses in the context of staging imaging (16%). The reason for the BM diagnosis was not recorded in the respective institutional databases for 519 patients.

**TABLE 1:** Study cohort characteristics.

Characteristic	*N* = 1431^a^
Primary tumor type	
Lung cancer	851 (59%)
Melanoma	328 (23%)
Breast cancer	252 (18%)
First surgery of BM	
Yes	1328 (93%)
No	94 (6.6%)
Unknown	9
Reason for BM diagnosis	
Symptomatic	747 (82%)
Staging	148 (16%)
Incidental	17 (1.9%)
Unknown	519
Extracranial metastases at time point of BM diagnosis	
Yes	660 (62%)
No	408 (38%)
Unknown	363
BM molecular profiling performed	
Yes	723 (51%)
No	708 (49%)

Abbreviation: BM, brain metastases.

^a^ *n* (%).

The most frequent PT was lung cancer (*n* = 851, 59%), followed by melanoma (*n* = 328, 23%) and breast cancer (*n* = 252, 18%). EM were present in 62% (*n* = 660) of cases at the time of BM diagnosis. Kaplan–Meier analysis of OS showed significantly different OS between BM from different PTs, with BM from breast cancer harboring the most favorable OS (median OS: 22.1 months), compared to 13.3 months for BM from melanoma and 11 months for BM from lung cancer (log‐rank statistic: *p* < 0.0001; see Figure S1).

### 3.2 BM Molecular Profiling Frequency and Characteristics

**FIGURE 1:**
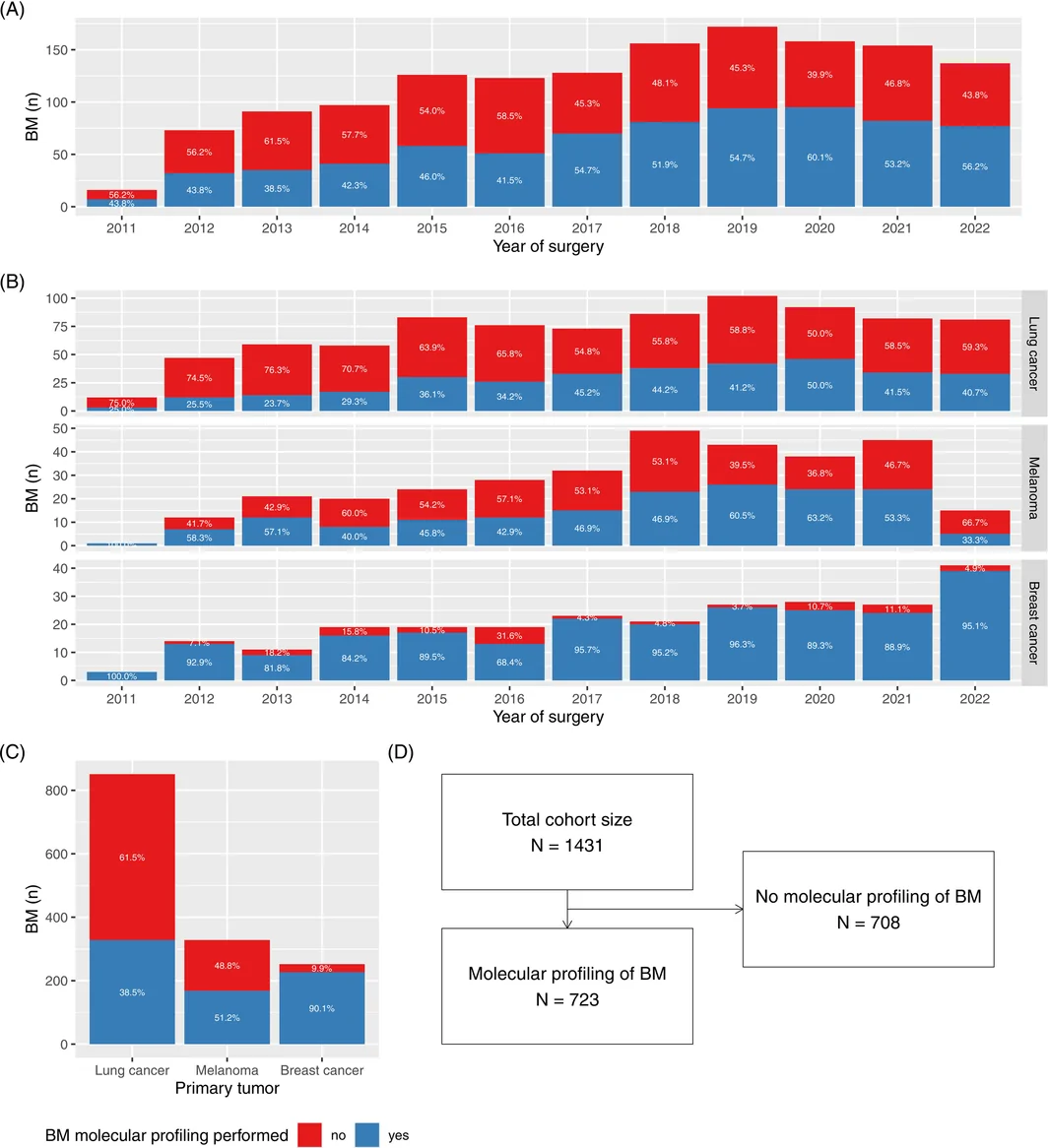
Study cohort and frequency of BM molecular profiling. (A–C) Bar plots showing the availability of BM molecular profiling per year of surgery (A) and primary tumor (B) and overall per primary tumor (C). (D) Inclusion flow chart of final study cohort. BM, brain metastases.

Depending on the PT and year of surgery, molecular profiling of BM tissue was performed at variable frequency (Figure 1A,B). Examining the year of surgery, there was an increasing trend in molecular profiling over the years, starting with less than 50% before 2017, peaking at up to 60% in 2020, and decreasing thereafter (Figure 1A). Molecular profiling was most frequently performed in breast cancer BM (90.1%), followed by melanoma BM (51.4%) and lung cancer BM (38.5%) (Figure 1C). Some institutional registries had incomplete records for 2022, which explains the discrepancies in those years (Figure 1A,B). Overall, of the 1431 BM, 723 BM (51%) had molecular profiling data performed and were included in the final study cohort for subsequent characterization (inclusion flow‐chart in Figure 1D). Kaplan–Meier analysis revealed significant differences in OS between the two groups, with longer OS in those with molecular profiling which was more pronounced in the subgroup of patients which were operated from 2017 onwards (median OS 19.3 vs. 9.9 months; *p* < 0.0001 for patients operated between 2017 and 2022; median OS 14.3 vs. 8.7 months for patients operated between 2011 and 2016; log‐rank statistic; Figure S2A).

### 3.3 Lung Cancer BM Molecular Profiling Characteristics

**FIGURE 2:**
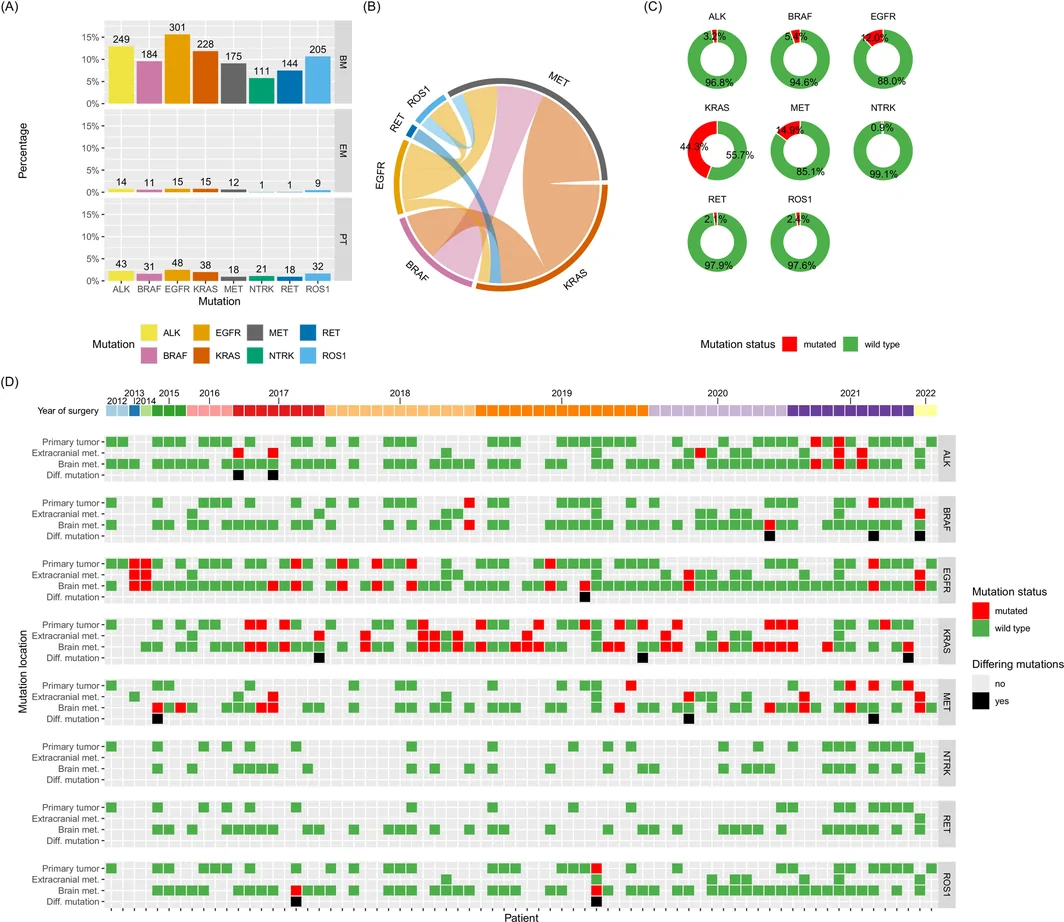
Lung cancer BM molecular profiling. Subset of patients with lung cancer in which molecular profiling of the BM was available. (A) Overall mutation characteristics with number of recorded genes which were assessed on BM, EM or PT tissue. (B) Chord diagram showing concurrently mutated genes in BM (only patients with at least two concurrently mutated genes are shown). (C) Donut chart showing the fraction of mutated/wildtype status per gene for BM. (D) Heatmap showing the mutational status and the BM and the corresponding PT/EM for each of the target genes. Differing mutations between BM and matched PT/EM are marked. BM, brain metastases; EM, extracranial metastases; PT, primary tumor.

For lung cancer BM with performed molecular profiling, the mutational status of the following genes was recorded for the BM and, if available, for any matched PT or EM tissue: ALK, BRAF, EGFR, FGFR, KRAS, MET, NTRK, RET, and ROS1. The mutational status of EGFR (*n* = 301 in BM, *n* = 47 in PT, and *n* = 15 in EM) was recorded most frequently, followed by ALK (*n* = 249 in BM, *n* = 42 in PT, and *n* = 14 in EM), and KRAS (*n* = 228 in BM, *n* = 37 in PT, and *n* = 15 in EM, Figure 2A). Co‐occurrences for mutated genes in BM were most frequently observed for KRAS/MET (Figure 2B for all co‐occurrences for all registered mutated BM genes). Overall, the percentage of mutated targets in BM was highest for KRAS (44.3%), followed by MET (14.9%) and EGFR (12%, Figure 2C).

For all patients where at least one matched PT/EM molecular profile was recorded (*n* = 72), we compared the respective mutational status to see if there were any differences between the BM and the PT/EM (see the heatmap in Figure 2D). Overall, 13 cases (18%) showed a differing status, most frequently with BRAF (*n* = 3), KRAS (*n* = 3), and MET (*n* = 3, Figure 2B).

### 3.4 Melanoma BM Molecular Profiling Characteristics

**FIGURE 3:**
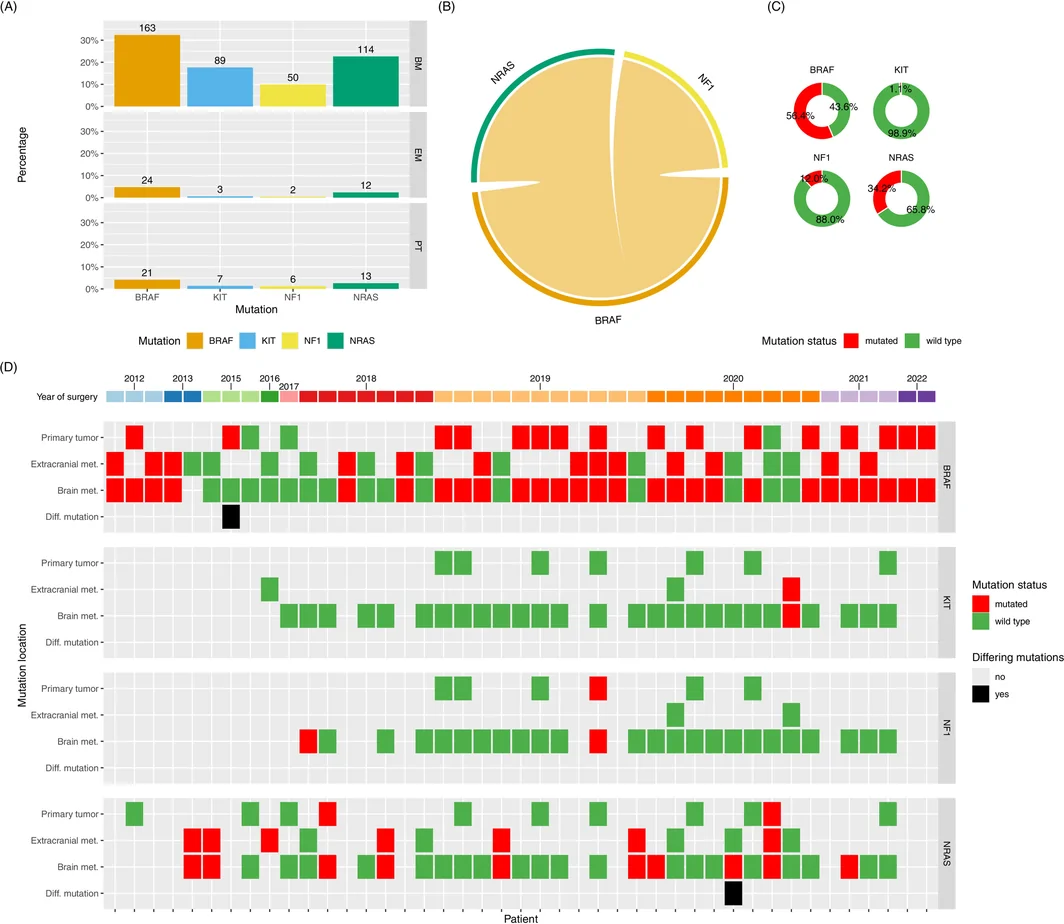
Melanoma BM molecular profiling. Subset of melanoma patients in which molecular profiling of the BM was available. (A) Overall mutation characteristics with number of recorded genes which were assessed on BM, EM or PT tissue. (B) Chord diagram showing concurrently mutated genes in BM (only patients with at least two concurrently mutated genes are shown). (C) Donut chart showing the fraction of mutated/wildtype status per gene for BM. (D) Heatmap showing the mutational status and the BM and the corresponding PT/EM for each of the target genes. Differing mutations between BM and matched PT/EM are marked. BM, brain metastases; EM, extracranial metastases; PT, primary tumor.

For melanoma BM with performed molecular profiling, the mutational status of the following genes was recorded for the BM and, if available, for any matched PT or EM tissue (see Section 2): BRAF, NRAS, KIT, and NF1. The BRAF status was recorded for 163 BM samples, 21 PT samples, and 24 EM samples; the NRAS status was recorded for 114 BM samples, 13 PT samples, and 12 EM samples, see Figure 3A. Co‐occurrences for mutated genes in BM were most frequently observed for NRAS/BRAF (Figure 3B). Overall, the percentage of mutated targets in BM was highest for BRAF (56.4%), followed by NRAS (34.2% see Figure 3C).

Co‐occurring BRAF and NRAS mutations were identified in the BM of three patients. Two had received prior BRAF inhibitor therapy, with one case showing a sub‐clonal NRAS mutation, consistent with therapy‐associated clonal evolution. In the third case, the co‐mutation was confirmed by re‐sequencing. NRAS mutation status was not assessed in the corresponding PTs, precluding comparison.

Of the cases with at least one matched PT/EM molecular profile (*n* = 43), two cases (4.7%) showed alternating mutational status (one BRAF and one NRAS; see Figure 3D).

### 3.5 Breast Cancer BM Molecular Profiling Characteristics

**FIGURE 4:**
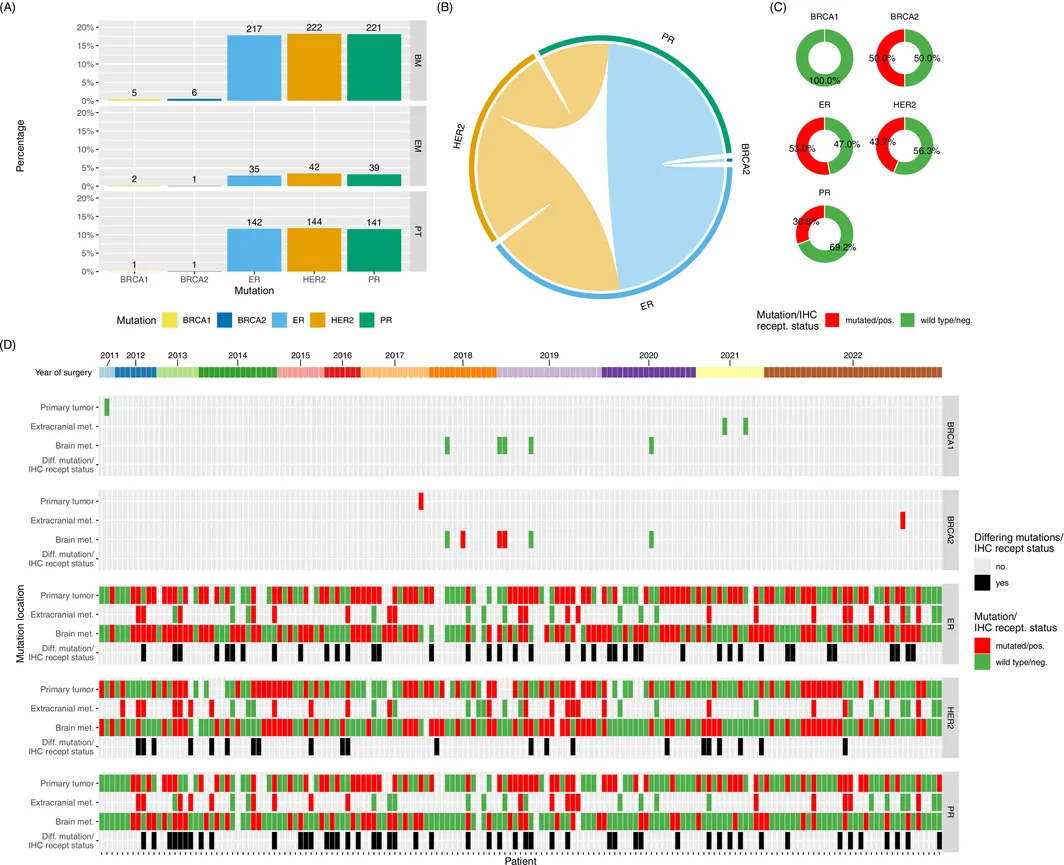
Breast cancer BM molecular/IHC receptor status profiling. Subset of breast cancer patients in which molecular/IHC profiling of the BM was available. (A) Overall mutation characteristics with number of recorded genes/hormone receptor markers which were assessed on BM, EM or PT tissue. (B) Chord diagram showing concurrently mutated genes/receptor status in BM (only patients with at least two concurrently mutated genes/pos. receptor status are shown). (C) Donut chart showing the fraction of mutated/wildtype (IHC receptor pos/neg) status per gene for BM. (D) Heatmap showing the mutational/IHC status and the BM and the corresponding PT/EM for each of the target genes/hormone receptors. Differing mutations/hormone receptor status between BM and matched PT/EM are marked. Mutational status “mutated” and “wildtype” are equivalent to IHC hormone receptor status “positive” and “negative.” BM, brain metastases; EM, extracranial metastases; PT, primary tumor; IHC, immunohistochemistry.

For breast cancer BM with performed profiling, the mutational and hormone/HER2 receptor status of the following genes/receptors were recorded for the BM and—if available—for any matched PT or EM tissue: ER, PR, HER2, BRCA1, BRCA2, and PIK3CA. Molecular status was most frequently analyzed of HER2 (*n* = 222 in BM, *n* = 144 in PT and *n* = 42 in EM), followed by PR (*n* = 221 in BM, *n* = 141 in PT and *n* = 39 in EM) and ER (*n* = 217 in BM, *n* = 142 in PT and *n* = 35 in EM) status was recorded (Figure 4A). Co‐occurrences for mutated genes/positive hormone receptor status in BM were most frequently observed for ER/PR and ER/HER2 (Figure 4B). Overall, the percentage of mutated/positive targets in BM was highest for ER (53%), followed by BRCA2 (50%), HER2 (43.7%) and PR (30.8%, see Figure 4C).

For the BM where at least one matched PT/EM molecular/IHC profiling was recorded (*n* = 161), 73 patients (45%) showed differing IHC hormone/HER2 status (41 ER, 50 PR and 22 HER2, see Figure 4D).

Survival analysis showed a trend to better OS in patients where a differing profile was detected between the BM and the PT/EM for patients operated from 2017 onwards without reaching the significant cutoff (median OS 28.6 vs. 17.7 months, *p* = 0.064 for patients operated 2017–2022; 14.1 vs. 14.4 months, *p* = 0.6 for patients operated 2011–2016; log‐rank statistic, see Figure S2B).

### 3.6 Change of Systemic Therapy Upon Molecular Profiling of BM

**FIGURE 5:**
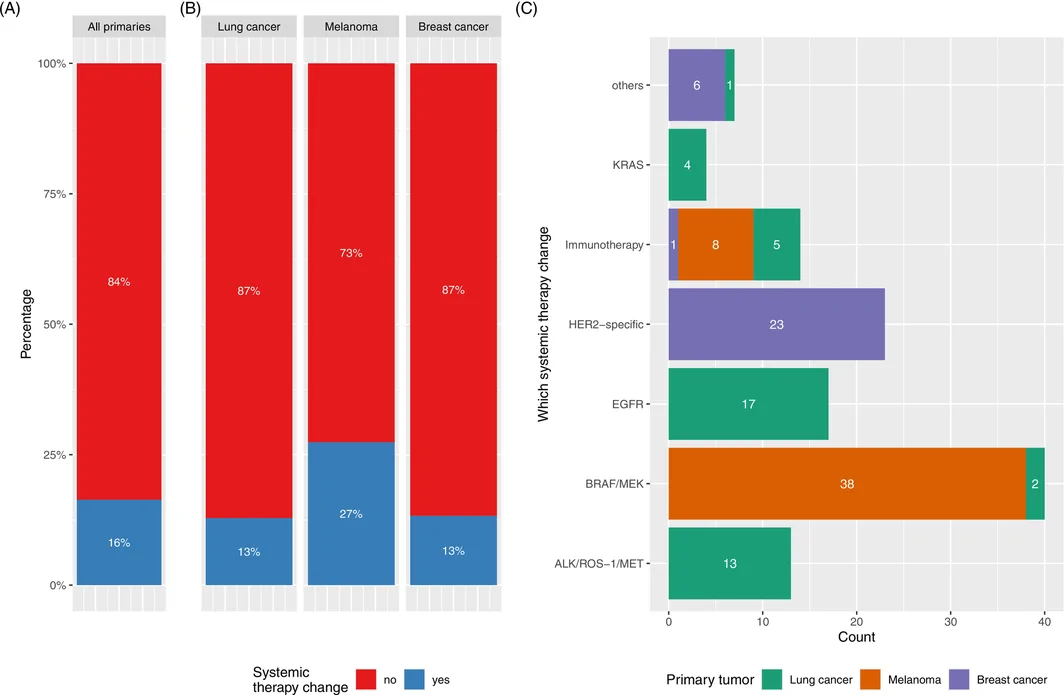
Overview of systemic therapy changes after BM molecular/IHC profiling. (A, B) Percentage of cases where systemic therapies were changed upon molecular profiling of BM, for the entire cohort (A) and for the different primary tumor subsets (B). (C) Systemic therapy classes which were administered, color‐coded by primary tumor type.

**FIGURE 6:**
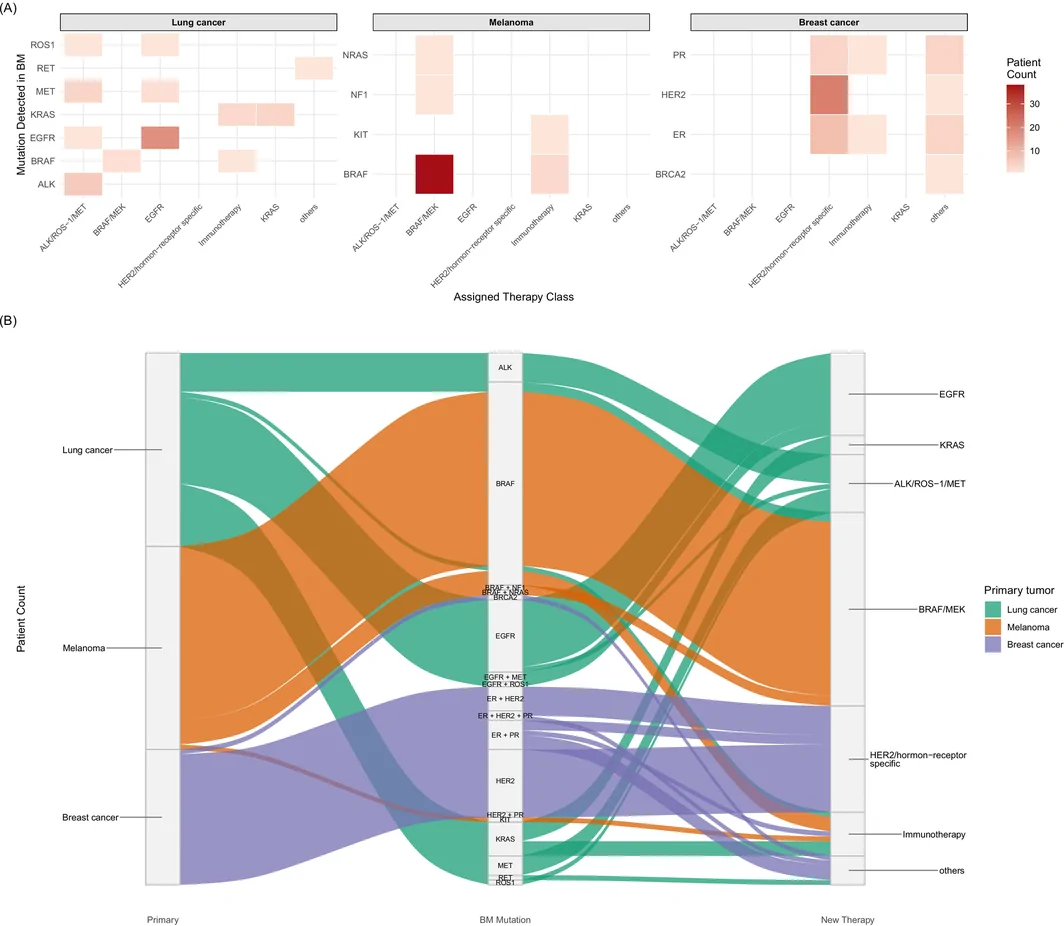
Systemic therapy changes after BM molecular/IHC profiling. (A) Heatmap showing the detected mutations in each BM type and the subsequent change in therapy class. (B) Sankey plot showing the primary tumor types and mutations detected in the BMs, as well as the subsequent new therapy for each patient for whom systemic therapy was adapted based on BM profiling. The plot is color‐coded by primary tumor type.

We next evaluated whether a change in systemic therapy was performed or anticipated based on molecular profiling of the BM in all cases where it was recorded. Overall, this occurred in 118 patients (16%) of cases with available molecular BM profiling (Figure 5A). This was most frequent in melanoma BM (27%, *n* = 46), followed by breast and lung cancer BM (both 13%, with *n* = 30 and *n* = 42 respectively; Figure 5B). When changes in systemic therapy were made, BRAF/MEK inhibitors were most frequently administered (*n* = 40, among these 38 with melanoma BM, including one with a previous exposition to BRAF/MEK inhibitors), followed by HER2‐specific therapies in breast cancer BM (*n* = 23, including 9 with a previous exposition to HER2 inhibitors) and EGFR inhibitors in lung cancer BM (*n* = 17, including one with a previous exposition to an EGFR inhibitor, Figure 5C). At the patient level, these therapy adaptations were highly concordant with the molecular findings in BM, with targeted treatments initiated in accordance with the respective alterations in the vast majority of cases (BRAF/MEK inhibition 40/40, EGFR inhibition 17/17, HER2‐directed therapy 20/23; see Figure 6).

Within our cohort, OS analysis showed longer OS in patients with breast cancer BM who underwent systemic therapy changes based on molecular/IHC profiling for patients operated between 2017 and 2022, compared to those without therapy change (median OS not reached vs. 26.1 months, *p* = 0.0085; Figure S2C). However, no difference in OS was observed in other tumor entities upon systemic therapy changes (Figure S2C). Lastly, univariate Cox Proportional‐Hazard model for OS showed that EM at the timepoint of BM diagnosis were negatively associated with OS (HR 1.27, 95% CI 1.04–1.54, *p* = 0.017) while breast cancer pathology being associated with longer OS compared to the other two entities (HR 0.7, 95% CI 0.56–0.87, *p* = 0.001, Figure S3). Furthermore, change in systemic therapies upon BM molecular profiling was also associated with better OS (HR 0.76, 95% CI 0.59–0.97, *p* = 0.03) as well as HER2 specific changes in systemic therapy (HR 0.28, 95% CI 0.12–0.68, *p* = 0.005, Figure S3).

## 4 Discussion

Molecular testing of BM can uncover actionable genetic alterations with the potential to guide systemic therapy and improve patient outcomes. Current clinical guidelines therefore recommend molecular profiling of resected BM, reflecting both the growing evidence for a role of oncogenic driver mutations and the increasing availability of targeted therapeutic approaches [20, 26]. This aligns with calls to expand molecular profiling in BM [16], as it may uncover therapeutic opportunities in cases of discordance with PTs or the emergence of resistance mechanisms while also providing prognostic insights. Therefore, it is essential to evaluate clinical practice in the context of current guidelines to generate evidence for improved patient outcomes, identify opportunities to improve oncologic care, determine the relevance of BM tissue sampling as a sole surgical indication, and ultimately inform the design of future clinical trials and the refinement of clinical guidelines.

Overall, our multicenter analysis revealed an upward trend in molecular testing rates over the past decade. Up to 60% of BM samples underwent genetic or IHC profiling. While this development reflects the growing availability of molecular profiling pipelines as well as targeted therapies and clinical awareness thereof, we noted a significant variation of molecular characterization between PT types and centers.

One rationale for molecular testing of BM is the possible divergence of targetable mutations in CNS metastasis compared to extracranial tumor manifestations. In addition, BM testing alone can support treatment decisions without the need for molecular data or divergent profiles of the PT or EM.

Since our study aimed to analyze “real‐life” daily practice with regard to molecular BM testing, both scenarios can be examined, with the respective limitations: The most frequent characterization of lung BM was observed for EGFR‐, ALK‐, and KRAS status which represent some of the most commonly targetable BM alterations [34]. In our cohort, discordances for these molecular targets in BM compared to extracranial sites were only noted scarcely, and reported discordance rates vary significantly for EGFR (0%–22.5%) [17, 35, 36] and KRAS (3.3%–39.4%) [37, 38, 39].

However, BM molecular profiling—irrespective of whether molecular information of extracranial tumor sites was available—informed systemic therapy changes in about 13% of patients, with therapy adaptations largely concordant with the respective molecular findings.

However, BM molecular profiling—irrespective of whether molecular information of extracranial tumor sites was available—informed systemic therapy adaptations in about 13% of patients, which were largely concordant with the respective molecular findings.

In melanoma, the introduction of immune checkpoint inhibition and targeted therapy led to a significant improvement in OS of eligible patients [10]. While the ideal combination of molecular agents is still under investigation, first studies show an advantage of concomitant application [24], supporting a more thorough BRAF‐testing regime upon BM diagnosis. Accordingly, we observed the highest testing rates in melanoma BM for BRAF‐mutations. This uncovered pathogenic alterations in 56%, which lies close to the reported mutation rates of 33%–52% in sequencing studies (52% [40], 42% [41], 33% [42]). Regarding BRAF, molecular profiling was associated with the use of matched targeted therapies in a substantial subset of patients. In real life, mutational differences between melanoma BM and their primary appeared less relevant for treatment planning than suggested by sequencing studies: in contrast to reported 14% discordance rates [18], BRAF status discordance was uncovered only in one case, as BM samples often presented the only source of molecular analysis. While NRAS‐ and NF1, and KIT‐mutated melanoma represent distinct genomic subtypes with potential therapeutic implications [43, 44], the lack of prospective clinical trials specifically in brain metastatic disease limits their application to clinical trials or off‐label treatment [21, 45]. This might explain the lower testing rates in our cohort, which can be expected to increase, as further evidence on their utility emerges [46, 47].

Breast cancer BM showed a high rate for receptor status analysis, which revealed BM/PT discordance in a substantial subset of patients. This is in accordance with the widely accepted relevance of subtype switching in breast cancer BM and reported conversion rates of overall 42.5% and 12% (8%–16%) of HER2 [48]. The described propensity of HER2‐gain in BM [49] was also evident in our cohort in 10 of the 22 patients where HER2 status showed discordance between BM and extracranial sites. Besides from receptor status, we observed a strikingly rare analysis of further genetic markers such as BRCA1/2 and PIK3CA, even though these are frequently mutated in breast cancer BM (BRCA1/2: 7.8%/5.5%, PIK3CA: 30.2%) and may be amenable to targeted treatment [50, 51]. This implementation gap might be due to their recent development and limited data on BM‐specific efficacy of approved PARP‐inhibitors such as olaparib and talazoparib [52], while a CNS‐penetrable PARP inhibitor, veliparib [53], and PI3K inhibitors are still tested in clinical trial for their efficacy in patients with BM (e.g., NCT05230810).

Our analysis demonstrates the clinical relevance of molecular profiling in resected BM; however, a substantial proportion of patients are not candidates for neurosurgical intervention. In this context, CSF‐based liquid biopsies, including ctDNA and circulating tumor cell‐based assays, have shown the ability to capture mutational profiles that are highly concordant with CNS metastases [54]. Although sequencing studies of selected driver genes in CSF samples report high diagnostic sensitivity [55, 56], these findings are largely derived from heterogeneous, NSCLC‐dominated datasets, often including leptomeningeal disease. Given that ctDNA extraction rates in intraparenchymal metastatic lesions are approximately 55% in clinical practice [54, 57, 58], CSF‐derived mutational profiles currently do not replace comprehensive analysis of available BM tissue, but rather represent a valuable adjunct, particularly in patients with non‐operative lesions or contraindications for surgery or for longitudinal assessment of treatment response and clonal evolution [56].

Overall, our multi‐institutional study demonstrated that, although molecular profiling of BM has increased in recent years, testing rates across all included tumor entities remained below 60%. Given the potential clinical impact—reflected by the proportion of patients in whom systemic therapy was adapted following BM profiling—this is unexpected, particularly as BM typically represents an advanced disease stage where comprehensive molecular testing could be of high relevance to uncover therapeutic opportunities. While our data indicate a potential survival benefit in a subset of patients who received systemic therapy modifications based on BM profiling, this observation requires confirmation in prospective studies that account for potential confounding factors.

### 4.1 Limitations

Due to the retrospective nature of the study there are several important limitations to consider: It represents a descriptive analysis of clinically available molecular and IHC data from surgically treated BM patients, rather than a comprehensive molecular characterization, and the findings may not be generalizable to the broader BM population. The retrospective design, predominance of resections over biopsies, and conduct in high‐volume tertiary centers with in‐house tumor boards introduce a selection bias. In addition, treatment decisions were not prospectively registered and cannot be solely attributed to BM molecular profiling but reflect multifactorial clinical decision‐making. Clinical outcome analyses were unadjusted and subject to confounding and case‐mix bias due to patient heterogeneity, comorbidities, treatment variability, and incomplete molecular data, limiting their interpretation to descriptive rather than causal associations.

## 5 Conclusions

In summary, our findings highlight the clinical value of molecular profiling in BM patients while exposing a critical gap between its potential and current real‐world implementation. Bridging this gap holds important implications and potential for multidisciplinary treatment teams and for BM patient outcomes.

## Author Contributions

**Maria Nikolaeva:** investigation, visualization, writing – original draft, writing – review and editing, formal analysis, data curation. **Jacopo Bellomo:** data curation, writing – review and editing, formal analysis. **Meltem Gönel:** data curation, writing – review and editing. **Vittorio Stumpo:** data curation, writing – review and editing. **Victor Egon Staartjes:** data curation, writing – review and editing. **Flavio Vasella:** data curation, writing – review and editing. **Kevin Akeret:** data curation, writing – review and editing. **André Sagerer:** data curation, writing – review and editing. **Anna Maria Zeitlberger:** data curation, writing – review and editing. **David Wasilewski:** data curation, writing – review and editing. **Christopher Krämer:** data curation, writing – review and editing. **Sven König:** data curation, writing – review and editing. **Julia Onken:** writing – review and editing. **Tareq Juratli:** writing – review and editing. **Alexander Köpp:** writing – review and editing. **Regina Reimann:** writing – review and editing. **Marcus Czabanka:** writing – review and editing. **Peter Vajkoczy:** writing – review and editing. **Ilker Y. Eyüpoglu:** writing – review and editing. **Oliver Bozinov:** writing – review and editing. **Michael Weller:** writing – review and editing. **Emilie Le Rhun:** writing – review and editing. **Luca Regli:** writing – review and editing. **Carlo Serra:** writing – review and editing. **Marian C. Neidert:** writing – review and editing, methodology, supervision. **Stefanos Voglis:** conceptualization, investigation, methodology, visualization, writing – original draft, writing – review and editing, formal analysis, supervision, data curation.

## Ethics Statement

The study was conducted in accordance with the local regulations and ethics committees of each institution. Patient registries were approved by the local ethics review boards (University Hospital Zurich identifier: 2017‐00093; Cantonal Hospital St. Gallen identifier: 2023‐01343; Charité University Hospital Berlin identifier: EA1/140/24, University Hospital Dresden identifier: EK 323122008, University Hospital Frankfurt identifier: SNO‐2‐2025).

## Conflicts of Interest

T.J. has received speaker honoraria from CSL‐Behring, AD board activities for Stryker, Ipsen, Servier. E.L.R. has received research grants from Bristol‐Myers Squibb, Servier and consulting fees from Astra Zeneca, Biodexa Sitoxi, Leo Pharma, medac, my tomorrows, Novartis, Pfizer, novocure, Seattle Genetics, Servier. M.W. has received research grants from Lundbeck and Novartis (to institution) and personal honoraria for lectures and advisory board participation or consulting from CureVac, Hemerion, Iqvia, Medac, Merck, My tomorrows, Novartis, novocure, Orbus, Philogen, Servier and Telix. M.C.N. has received research grants from Novocure and honoraria for consulting or lectures from WISE, MSD, Osteopore, Servier and Terumo. The other authors declare no conflicts of interest.

## Supporting information


**Table S1:** Molecular profiling assays for BM tissue.
**Table S2:** Detected molecular alterations in BMs.
**Figure S1:** Overall survival analysis of the entire cohort.
**Figure S2:** Overall survival analysis dichotomized by year of surgery.
**Figure S3:** Cox proportional hazards model of OS.

## Data Availability

Anonymized patient data, analysis scripts, and further information that support the findings of this study are available from the corresponding author upon reasonable request.

## References

[1] L. Nayak , E. Q. Lee , and P. Y. Wen , “Epidemiology of Brain Metastases,” Current Oncology Reports 14, no. 1 (2012): 48–54, 10.1007/s11912-011-0203-y. 22012633

[2] A. S. Achrol , R. C. Rennert , C. Anders , et al., “Brain Metastases,” Nature Reviews Disease Primers 5, no. 1 (2019): 1–26. 10.1038/s41572-018-0055-y 30655533

[3] A. Steindl , T. J. Brunner , K. Heimbach , et al., “Changing Characteristics, Treatment Approaches and Survival of Patients With Brain Metastasis: Data From Six Thousand and Thirty‐One Individuals Over an Observation Period of 30 Years,” European Journal of Cancer 162 (2022): 170–181, 10.1016/j.ejca.2021.12.005. 34998049

[4] O. E. Yri , G. L. Astrup , A. T. Karlsson , et al., “Survival and Quality of Life After First‐Time Diagnosis of Brain Metastases: A Multicenter, Prospective, Observational Study,” Lancet Regional Health 49 (2025): 101181, 10.1016/j.lanepe.2024.101181. PMC11728971 39807153

[5] V. Stumpo , A. Carretta , J. Bellomo , et al., “Surgical Tumor Volume Reduction in Patients With Brain Metastases: A Systematic Review and Meta‐Analysis,” Cancer Treatment Reviews 139 (2025): 102981, 10.1016/j.ctrv.2025.102981. 40554919

[6] L. Padevit , A. M. Zeitlberger , N. Maldaner , et al., “Multiple Craniotomies in Patients With Brain Metastases: A Two‐Center, Propensity Score‐Matched Study,” Neurosurgical Review 47, no. 1 (2024): 354, 10.1007/s10143-024-02578-8. PMC11281945 39060536

[7] J. Bellomo , A. M. Zeitlberger , L. Padevit , et al., “Role of Microsurgical Tumor Burden Reduction in Patients With Breast Cancer Brain Metastases Considering Molecular Subtypes: A Two‐Center Volumetric Survival Analysis,” Journal of Neuro‐Oncology 169, no. 2 (2024): 379–390, 10.1007/s11060-024-04728-w. PMC11341656 38829577

[8] S. Voglis , L. Padevit , C. H. B. van Niftrik , et al., “Safety of Microneurosurgical Interventions for Superficial and Deep‐Seated Brain Metastases: Single‐Center Cohort Study of 637 Consecutive Cases,” Journal of Neuro‐Oncology 165, no. 2 (2023): 271–278, 10.1007/s11060-023-04478-1. PMC10689541 37945819

[9] S. Voglis , V. Schaller , T. Müller , et al., “Maximal Surgical Tumour Load Reduction in Immune‐Checkpoint Inhibitor Naïve Patients With Melanoma Brain Metastases Correlates With Prolonged Survival,” European Journal of Cancer 175 (2022): 158–168, 10.1016/j.ejca.2022.08.020. 36126476

[10] J. P. Merola , J. Ocen , S. Kumar , J. Powell , and C. Hayhurst , “Survival in Melanoma Brain Metastases in the Era of Novel Systemic Therapies,” Neuro‐Oncology Advances 2, no. 1 (2020): vdaa144, 10.1093/noajnl/vdaa144. PMC7764504 33392503

[11] H. A. Tawbi , P. A. Forsyth , F. S. Hodi , et al., “Long‐Term Outcomes of Patients With Active Melanoma Brain Metastases Treated With Combination Nivolumab Plus Ipilimumab (CheckMate 204): Final Results of an Open‐Label, Multicentre, Phase 2 Study,” Lancet Oncology 22, no. 12 (2021): 1692–1704, 10.1016/S1470-2045(21)00545-3. PMC9328029 34774225

[12] H. A. Tawbi , P. A. Forsyth , A. Algazi , et al., “Combined Nivolumab and Ipilimumab in Melanoma Metastatic to the Brain,” New England Journal of Medicine 379, no. 8 (2018): 722–730, 10.1056/NEJMoa1805453. PMC8011001 30134131

[13] J. Wang and H. A. Tawbi , “Emergent Immunotherapy Approaches for Brain Metastases,” Neuro‐Oncology Advances 3, no. Supplement_5 (2021): v43–v51, 10.1093/NOAJNL/VDAB138. PMC8633738 34859232

[14] M. H. Bailey , C. Tokheim , E. Porta‐Pardo , et al., “Comprehensive Characterization of Cancer Driver Genes and Mutations,” Cell 173, no. 2 (2018): 371–385.e18, 10.1016/j.cell.2018.02.060. PMC6029450 29625053

[15] P. K. Brastianos , S. L. Carter , S. Santagata , et al., “Genomic Characterization of Brain Metastases Reveals Branched Evolution and Potential Therapeutic Targets,” Cancer Discovery 5, no. 11 (2015): 1164–1177, 10.1158/2159-8290.CD-15-0369. PMC4916970 26410082

[16] E. Shen , A. E. D. Van Swearingen , M. J. Price , et al., “A Need for More Molecular Profiling in Brain Metastases,” Frontiers in Oncology 11 (2021): 785064, 10.3389/fonc.2021.785064. PMC8821807 35145903

[17] D. Li , Y. Huang , L. Cai , et al., “Genomic Landscape of Metastatic Lung Adenocarcinomas From Large‐Scale Clinical Sequencing,” Neoplasia 23, no. 12 (2021): 1204–1212, 10.1016/j.neo.2021.10.001. PMC8571538 34735995

[18] E. J. Hannan , D. P. O'Leary , S. P. MacNally , et al., “The Significance of BRAF V600E Mutation Status Discordance Between Primary Cutaneous Melanoma and Brain Metastases: The Implications for BRAF Inhibitor Therapy,” Medicine (Baltimore) 96, no. 48 (2017): e8404, 10.1097/MD.0000000000008404. PMC5728729 29310328

[19] J. Jung , S. H. Lee , M. Park , et al., “Discordances in ER, PR, and HER2 Between Primary Breast Cancer and Brain Metastasis,” Journal of Neuro‐Oncology 137, no. 2 (2018): 295–302, 10.1007/s11060-017-2717-0. PMC5851692 29260362

[20] K. Huntoon , J. B. Elder , G. Finger , et al., “Congress of Neurological Surgeons Systematic Review and Evidence‐Based Guidelines Update for the Role of Emerging Therapies in the Management of Patients With Metastatic Brain Tumors,” Neurosurgery 96 (2025): 1172–1177, 10.1227/neu.0000000000003383. 40094364

[21] C. Garbe , T. Amaral , K. Peris , et al., “European Consensus‐Based Interdisciplinary Guideline for Melanoma. Part 2: Treatment ‐ Update 2024,” European Journal of Cancer 215 (2025): 115153, 10.1016/j.ejca.2024.115153. 39709737

[22] G. V. Long , V. Atkinson , S. N. Lo , et al., “Ipilimumab Plus Nivolumab Versus Nivolumab Alone in Patients With Melanoma Brain Metastases (ABC): 7‐Year Follow‐Up of a Multicentre, Open‐Label, Randomised, Phase 2 Study,” Lancet Oncology 26, no. 3 (2025): 320–330, 10.1016/S1470-2045(24)00735-6. 39978375

[23] M. A. Davies , P. Saiag , C. Robert , et al., “Dabrafenib Plus Trametinib in Patients With BRAFV600‐Mutant Melanoma Brain Metastases (COMBI‐MB): A Multicentre, Multicohort, Open‐Label, Phase 2 Trial,” Lancet Oncology 18, no. 7 (2017): 863–873, 10.1016/S1470-2045(17)30429-1. PMC5991615 28592387

[24] R. Dummer , P. Queirolo , P. Gerard Duhard , et al., “Atezolizumab, Vemurafenib, and Cobimetinib in Patients With Melanoma With CNS Metastases (TRICOTEL): A Multicentre, Open‐Label, Single‐Arm, Phase 2 Study,” Lancet Oncology 24, no. 12 (2023): e461–e471, 10.1016/S1470-2045(23)00334-0. 37459873

[25] SWOG Cancer Research Network , “A Randomized Phase 2 Trial of Encorafenib + Binimetinib + Nivolumab vs Ipilimumab + Nivolumab in BRAF‐V600 Mutant Melanoma With Brain Metastases,” clinicaltrials.gov, 2025, https://clinicaltrials.gov/study/NCT04511013.

[26] E. L. Rhun , M. Guckenberger , M. Smits , et al., “EANO–ESMO Clinical Practice Guidelines for Diagnosis, Treatment and Follow‐Up of Patients With Brain Metastasis From Solid Tumours☆,” Annals of Oncology 32, no. 11 (2021): 1332–1347, 10.1016/j.annonc.2021.07.016. 34364998

[27] M. A. Vogelbaum , P. D. Brown , H. Messersmith , et al., “Treatment for Brain Metastases: ASCO‐SNO‐ASTRO Guideline,” Journal of Clinical Oncology 40, no. 5 (2022): 492–516, 10.1200/JCO.21.02314. 34932393

[28] J. R. Conway , J. L. Warner , W. S. Rubinstein , and R. S. Miller , “Next‐Generation Sequencing and the Clinical Oncology Workflow: Data Challenges, Proposed Solutions, and a Call to Action,” JCO Precision Oncology 3 (2019): PO.19.00232, 10.1200/PO.19.00232. PMC7446333 32923847

[29] K. H. Allison , M. E. H. Hammond , M. Dowsett , et al., “Estrogen and Progesterone Receptor Testing in Breast Cancer: ASCO/CAP Guideline Update,” Journal of Clinical Oncology 38, no. 12 (2020): 1346–1366, 10.1200/JCO.19.02309. 31928404

[30] A. C. Wolff , M. E. H. Hammond , K. H. Allison , et al., “Human Epidermal Growth Factor Receptor 2 Testing in Breast Cancer: American Society of Clinical Oncology/College of American Pathologists Clinical Practice Guideline Focused Update,” Archives of Pathology & Laboratory Medicine 142, no. 11 (2018): 1364–1382, 10.5858/arpa.2018-0902-SA. 29846104

[31] V. A. Venur , J. V. Cohen , and P. K. Brastianos , “Targeting Molecular Pathways in Intracranial Metastatic Disease,” Frontiers in Oncology 9 (2019): 99, 10.3389/fonc.2019.00099. PMC6409309 30886831

[32] R Core Team , “R: A Language and Environment for Statistical Computing,” 2024, https://www.r‐project.org/.

[33] Team Rs , “RStudio: Integrated Development for R,” 2020.

[34] R. Soffietti , M. Ahluwalia , N. Lin , and R. Ruda , “Management of Brain Metastases According to Molecular Subtypes,” Nature Reviews. Neurology 16, no. 10 (2020): 557–574, 10.1038/s41582-020-0391-x. 32873927

[35] K. M. Kim , S. H. Lee , S. M. Kim , et al., “Discordance of Epidermal Growth Factor Receptor Mutation Between Brain Metastasis and Primary Non‐Small Cell Lung Cancer,” Brain Tumor Research and Treatment 7, no. 2 (2019): 137–140, 10.14791/btrt.2019.7.e44. PMC6829080 31686445

[36] W. Zhao , W. Zhou , L. Rong , et al., “Epidermal Growth Factor Receptor Mutations and Brain Metastases in Non‐Small Cell Lung Cancer,” Frontiers in Oncology 12 (2022): 912505, 10.3389/fonc.2022.912505. PMC9707620 36457515

[37] K. M. Rau , H. K. Chen , L. Y. Shiu , et al., “Discordance of Mutation Statuses of Epidermal Growth Factor Receptor and K‐Ras Between Primary Adenocarcinoma of Lung and Brain Metastasis,” International Journal of Molecular Sciences 17, no. 4 (2016): 524, 10.3390/ijms17040524. PMC4848980 27070580

[38] R. Tonse , M. Rubens , H. Appel , et al., “Systematic Review and Meta‐Analysis of Lung Cancer Brain Metastasis and Primary Tumor Receptor Expression Discordance,” Discover Oncology 12, no. 1 (2021): 48, 10.1007/s12672-021-00445-2. PMC8777541 35201504

[39] H. Wang , Q. Ou , D. Li , et al., “Genes Associated With Increased Brain Metastasis Risk in Non‐Small Cell Lung Cancer: Comprehensive Genomic Profiling of 61 Resected Brain Metastases Versus Primary Non‐Small Cell Lung Cancer (Guangdong Association Study of Thoracic Oncology 1036),” Cancer 125, no. 20 (2019): 3535–3544, 10.1002/cncr.32372. 31287555

[40] G. K. In , K. Poorman , M. Saul , et al., “Molecular Profiling of Melanoma Brain Metastases Compared to Primary Cutaneous Melanoma and to Extracranial Metastases,” Oncotarget 11, no. 33 (2020): 3118–3128, 10.18632/oncotarget.27686. PMC7443369 32913556

[41] R. Rabbie , P. Ferguson , K. Wong , et al., “The Mutational Landscape of Melanoma Brain Metastases Presenting as the First Visceral Site of Recurrence,” British Journal of Cancer 124, no. 1 (2021): 156–160, 10.1038/s41416-020-01090-2. PMC7782512 33024263

[42] H. N. Vasudevan , C. Delley , W. C. Chen , et al., “Molecular Features of Resected Melanoma Brain Metastases, Clinical Outcomes, and Responses to Immunotherapy,” JAMA Network Open 6, no. 8 (2023): e2329186, 10.1001/jamanetworkopen.2023.29186. PMC10436135 37589977

[43] Cancer Genome Atlas Network , “Genomic Classification of Cutaneous Melanoma,” Cell 161, no. 7 (2015): 1681–1696, 10.1016/j.cell.2015.05.044. PMC4580370 26091043

[44] C. Saberian , P. Sperduto , and M. A. Davies , “Targeted Therapy Strategies for Melanoma Brain Metastasis,” Neuro‐Oncology Advances 3, no. Supplement_5 (2021): v75–v85, 10.1093/NOAJNL/VDAB131. PMC8633745 34859235

[45] J. Mangana , B. C. Özdemir , D. Mihic‐Probst , et al., “The Updated Swiss Guidelines for the Treatment and Follow‐Up of Cutaneous Melanoma,” Swiss Medical Weekly 155 (2025): 4210, 10.57187/s.4210. 40811146

[46] J. Larkin , R. Marais , N. Porta , et al., “Nilotinib in KIT‐Driven Advanced Melanoma: Results From the Phase II Single‐Arm NICAM Trial,” Cell Reports Medicine 5, no. 3 (2024): 101435, 10.1016/j.xcrm.2024.101435. PMC10982988 38417447

[47] M. Salzmann , J. Pawlowski , C. Loquai , et al., “MEK Inhibitors for Pre‐Treated, NRAS‐Mutated Metastatic Melanoma: A Multi‐Centre, Retrospective Study,” European Journal of Cancer 166 (2022): 24–32, 10.1016/j.ejca.2022.02.008. 35272084

[48] R. Kotecha , R. Tonse , M. Rubens , et al., “Systematic Review and Meta‐Analysis of Breast Cancer Brain Metastasis and Primary Tumor Receptor Expression Discordance,” Neuro‐Oncology Advances 3, no. 1 (2021): vdab010, 10.1093/noajnl/vdab010. PMC8055057 33898990

[49] A. F. C. Hulsbergen , A. Claes , V. K. Kavouridis , et al., “Subtype Switching in Breast Cancer Brain Metastases: A Multicenter Analysis,” Neuro‐Oncology 22, no. 8 (2020): 1173–1181, 10.1093/neuonc/noaa013. PMC7471502 31970416

[50] A. Giannoudis , E. S. Sokol , T. Bhogal , et al., “Breast Cancer Brain Metastases Genomic Profiling Identifies Alterations Targetable by Immune‐Checkpoint and PARP Inhibitors,” npj Precision Oncology 8, no. 1 (2024): 282, 10.1038/s41698-024-00761-0. PMC11662007 39706915

[51] A. J. Morgan , A. Giannoudis , and C. Palmieri , “The Genomic Landscape of Breast Cancer Brain Metastases: A Systematic Review,” Lancet Oncology 22, no. 1 (2021): e7–e17, 10.1016/S1470-2045(20)30556-8. 33387511

[52] S. Jusino , C. E. Fadul , and P. Dillon , “Systematic Review of the Management of Brain Metastases From Hormone Receptor Positive Breast Cancer,” Journal of Neuro‐Oncology 162, no. 1 (2023): 45–57, 10.1007/s11060-023-04276-9. PMC10049940 36884200

[53] V. Diéras , H. S. Han , B. Kaufman , et al., “Veliparib With Carboplatin and Paclitaxel in BRCA‐Mutated Advanced Breast Cancer (BROCADE3): A Randomised, Double‐Blind, Placebo‐Controlled, Phase 3 Trial,” Lancet Oncology 21, no. 10 (2020): 1269–1282, 10.1016/S1470-2045(20)30447-2. 32861273

[54] J. Wu , Z. Liu , T. Huang , et al., “Cerebrospinal Fluid Circulating Tumor DNA Depicts Profiling of Brain Metastasis in NSCLC,” Molecular Oncology 17, no. 5 (2023): 810–824, 10.1002/1878-0261.13357. PMC10158766 36495130

[55] O. O. Olayode , B. T. Ogunoye , E. O. Oladeji , O. E. Olayinka , and T. J. Oladosu , “Cerebrospinal Fluid Circulating Tumor DNA (ctDNA) as a Biomarker for CNS Metastases in Non‐Small Cell Lung Cancer (NSCLC): A Systematic Review and Meta‐Analysis Comparing CSF ctDNA and Traditional Methods,” BMC Cancer 25, no. 1 (2025): 1246, 10.1186/s12885-025-14583-1. PMC12312583 40739228

[56] S. D. Robinson , J. de Boisanger , F. M. G. Pearl , G. Critchley , N. Rosenfelder , and G. Giamas , “A Brain Metastasis Liquid Biopsy: Where Are We Now?,” Neuro‐Oncology Advances 6, no. 1 (2024): vdae066, 10.1093/noajnl/vdae066. PMC11102938 38770219

[57] R. A. Hickman , A. M. Miller , B. M. Holle , et al., “Real‐World Experience With Circulating Tumor DNA in Cerebrospinal Fluid From Patients With Central Nervous System Tumors,” Acta Neuropathologica Communications 12, no. 1 (2024): 151, 10.1186/s40478-024-01846-4. PMC11406943 39289779

[58] A. Köpp , P. Westarp , M. Nowak , et al., “Targeted Next‐Generation Sequencing‐Based Sequencing of Cell‐Free DNA in Cerebrospinal Fluid Uncovers Cancer‐Specific Mutations in Patients With Brain Cancer Using a Widely Available Panel,” Neuro‐Oncology Advances 8, no. 1 (2026): vdaf270, 10.1093/noajnl/vdaf270. PMC12883210 41664825

