# Supplementary material for: Clinical Implementation and Oncological Relevance of Molecular Profiling in Brain Metastases Patients—A Multicenter Retrospective Cohort Study
Source: Int J Cancer. 2026 Jun 18;159(10):2562–74. doi: 10.1002/ijc.70611 (PMC13595484; doi:10.1002/ijc.70611)
Supplement: Supplementary file 1 [file IJC-159-2562-s001.pdf]

Clinical Implementation and Oncological Relevance of Molecular Profiling in Brain Metastases Patients - A Multicenter Retrospective Cohort Study

Maria Nikolaeva, Jacopo Bellomo, Meltem Gönel, Vittorio Stumpo, Victor Egon Staartjes, Flavio Vasella, Kevin Akeret, André Sagerer, Anna Maria Zeitlberger, David Wasilewski, Christopher Krämer, Sven König, Julia Onken, Tareq Juratli, Alexander Köpp, Regina Reimann, Marcus Czabanka, Peter Vajkoczy, Ilker Y Eyüpoglu, Oliver Bozinov, Michael Weller, Emilie Le Rhun, Luca Regli, Carlo Serra, Marian C Neidert, Stefanos Voglis

Table of contents:

- Supplementary tables

Supplementary Table 1: Molecular profiling assays for BM tissue
Supplementary Table 2: Detected molecular alterations in BMs

- Supplementary figures

Supplementary Figure 1: Overall survival analysis of the entire cohort
Supplementary Figure 2: Overall survival analysis dichotomized by year of surgery
Supplementary Figure 3: Cox proportional hazards model of OS

Center	Assays
Berlin	FISH (ALK, ROS1)<br>TruSight™ Oncology 500 Assay
Frankfurt	FISH (ALK, RET, ROS1, MET)<br>Pyrosequencing (EGFR, KRAS, NRAS, BRAF)<br>NGS Custom Lung-Panel (GeneRead DNSseq Qiagen)<br>NGS GeneRead DNSseq Panels nNGM*-v1 and nNGM-v2 (Qiagen)<br>NGS Oncomine Comprehensive Assay v3 (ThermoFisher)<br>NGS Ion AmpliSeq, nNGM-Panel-v2 (ThermoFisher)<br>NGS RNA ARCHER FusionPlex Lung (Integrated DNA Technologies IDT)<br>NGS TruSight Oncology 500 Panel (Illumina)<br>HER2-CISH/SISH<br>IHC (HER2, PR, ER)
Dresden	FISH (ALK, ROS, MET)<br>PCR-Assay (EGFR)<br>Pyrosequencing (NRAS, BRAF)<br>NGS QIAseq Targeted DNA Panels nNGM-v1 and nNGM-v2 (Qiagen)<br>NGS TruSight Tumorpanels TST15/TST26 (Illumina)<br>NGS QIAseq Targeted RNA Scan Panel (Qiagen)<br>NGS Human Actionable Solid Tumor Panel (Qiagen)<br>IHC (HER2, ER, PR)
St.Gallen	FISH (ALK, ROS1, HER2)<br>PCR-Assay (EGFR)<br>NGS Oncomine Comprehensive Assay v3 (Ion Torrent)<br>IHC (HER2, ER, PR)
Zurich	FISH (ALK, HER2, MET, RET, ROS1)<br>PCR-Assay (BRAF, NRAS)<br>Sequencing Sanger/Pyro (BRAF, EGFR, ERBB2, KIT, KRAS, NRAS)<br>NGS Oncomine Focus Assay (ThermoFisher)<br>NGS FoundationOne CDx (Foundation Medicine)<br>NGS MelArray (Roche Nimblegen)<br>IHC (HER2, ER, PR)
	*Panel based on collaborative network "national Network Genomic Medicine (nNGM)"

Supplementary Table 1: Molecular profiling assays for BM tissue
Summary of the various assays utilized across participating centers to identify molecular alterations in resected BM samples.

FISH Fluorescence In Situ Hybridization; NGS Next Generation Sequencing; IHC Immunohistochemistry; PCR Polymerase Chain Reaction

Lung cancer	N	Melanoma	N	Breast cancer	N
BRAF amplification	3	BRAF.V600E	68	HER2 overexpression (IHC/FISH Amp/ERBB2 Amp)	98
BRAF.V600E	1	BRAF.V600K	15	BRCA2 truncating mutation	2
BRAF Non-V600_missense	4	BRAF Non-v600_Missense	3	BRCA2 VUS	1
BRAF not defined	2	BRAF ot defined	9		
ALK EML4-ALK Fusion	5	NRAS.Q61	35		
ALK no defined (FISH, complex rearrangements)	3	NRAS.G12	1		
ALK Missense	1	NRAS.G13	1		
EGFR L858R	9	NF1 Nonsense/Frameshift/ Splice site mutation	4		
EGFR exon 19 deletion	13	NF1 Missense	2		
EGFR amplification	4	KIT Ampilfication	1		
EGFR exon 20 insertion	3				
EGFR others (VUS, not defined)	7				
KRAS G12C	37				
KRAS G12V	21				
KRAS G12D	8				
KRAS G12 others	16				
KRAS G13	12				
KRAS others (incl. Q61, amplification, VUS, not defined)	15				
MET Exon 14 Skipping Mutation	7				
MET Amplification	13				
MET others (VUS, not defined)	8				
ROS1 fusions	3				
ROS1 others (VUS, not defined)	3				
RET fusion	1				
RET others (VUS, not defined)	2				
NTRK Amplification	1				

Supplementary Table 2: Detected molecular alterations in BMs
Summary of the various detected mutations with the respective protein changes as recorded in the study cohort
VUS variants of uncertain significance

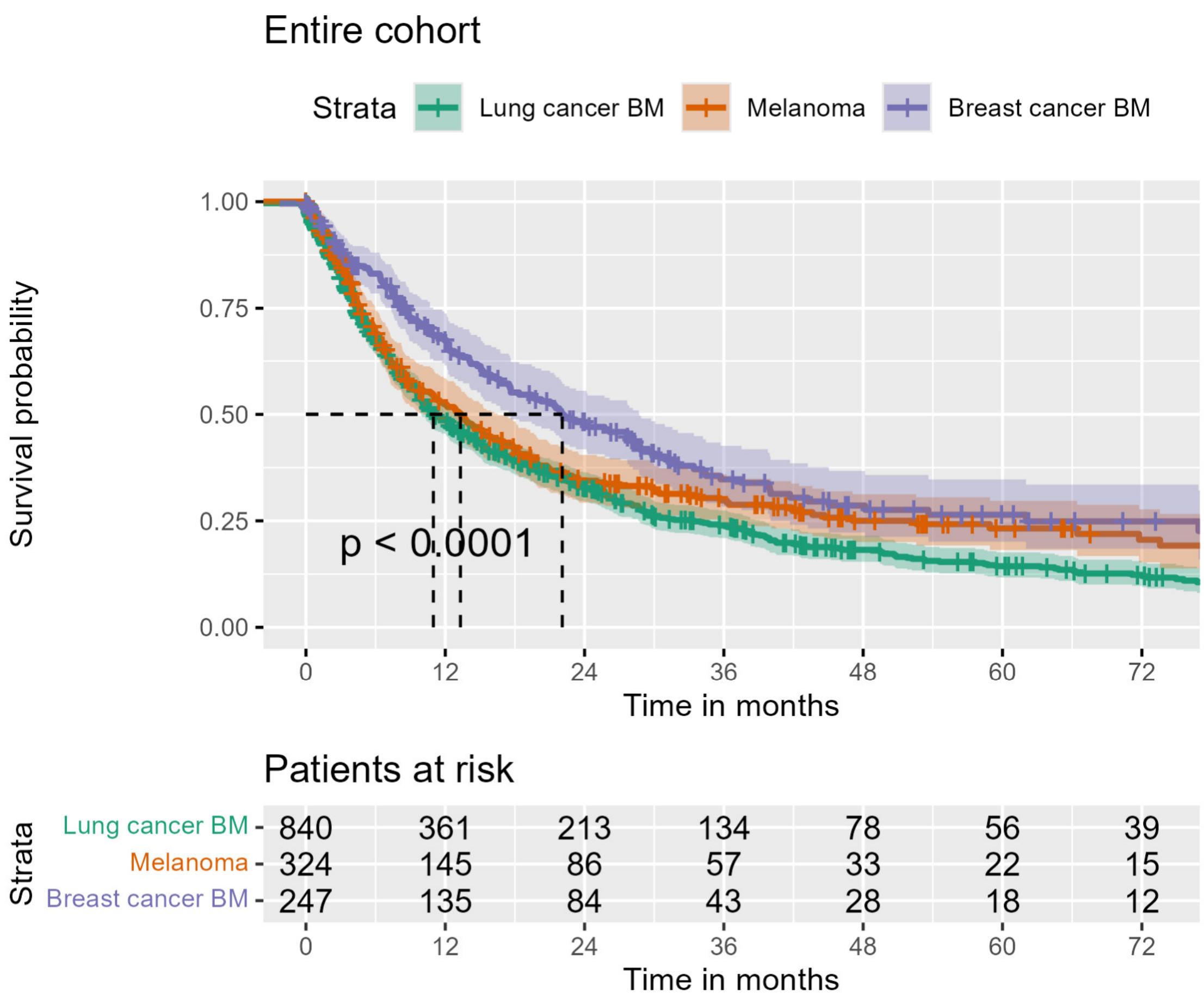

Supplementary Figure 1: Overall survival analysis of the entire cohort.
Kaplan-Meier curves for OS of the entire cohort stratified by primary tumor type. Log-rank statistics.

BM brain metastases; OS overall survival.

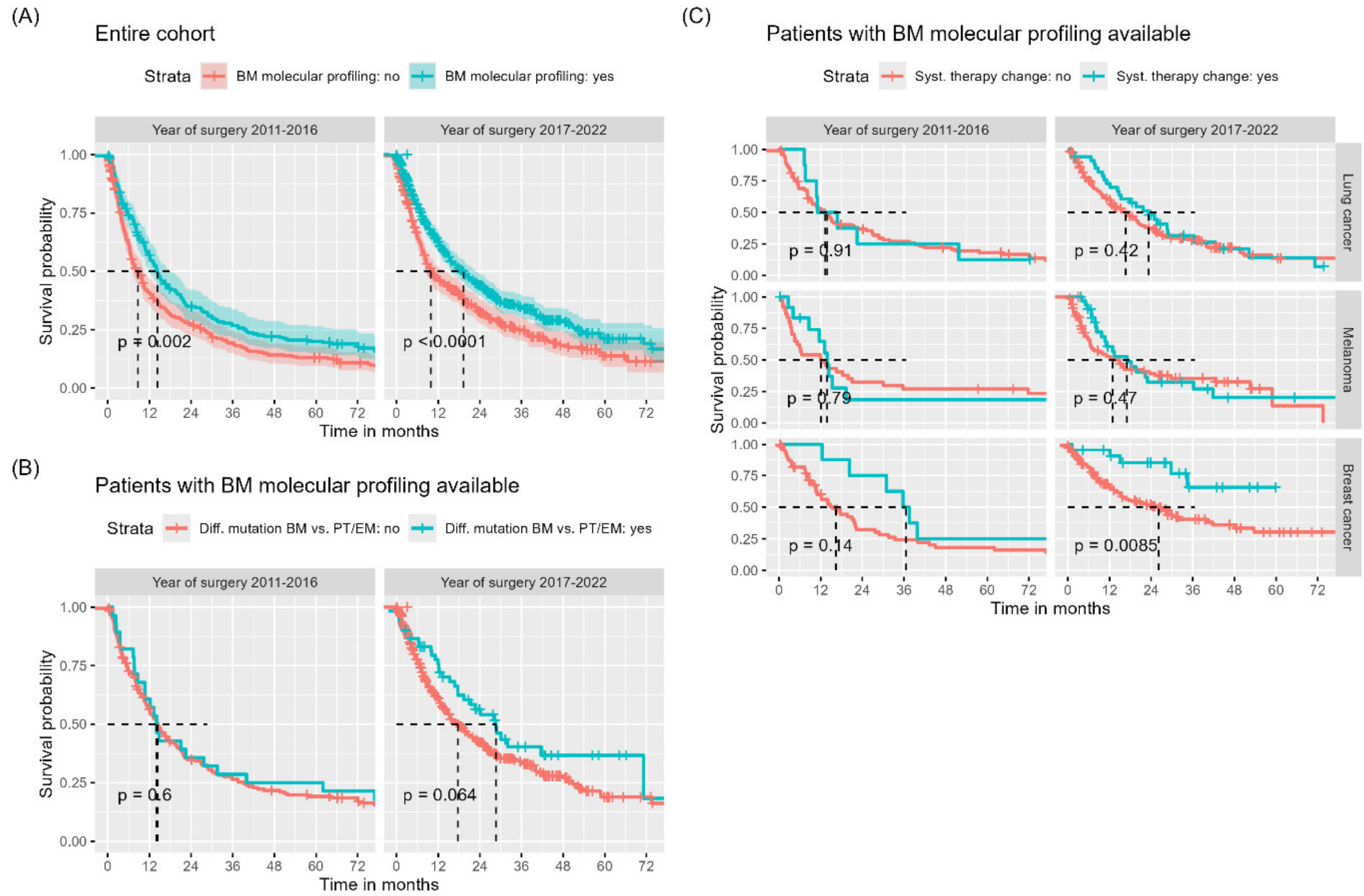

Supplementary Figure 2: Overall survival analysis dichotomized by year of surgery. Kaplan-Meier curves for OS of the cohort dichotomized based on the year of surgery (2011-2016 vs. 2017-2022), (A) stratified by whether molecular profiling of BM was performed, (B) for cases where different mutational/IHC (hormone) receptor profiles were detected between BM and PT/EM (C) and for the change in systemic therapies upon BM profiling stratified by primary tumor histology. Log-rank statistics.

BM brain metastases; PT primary tumor; EM extracranial metastases; OS overall survival.

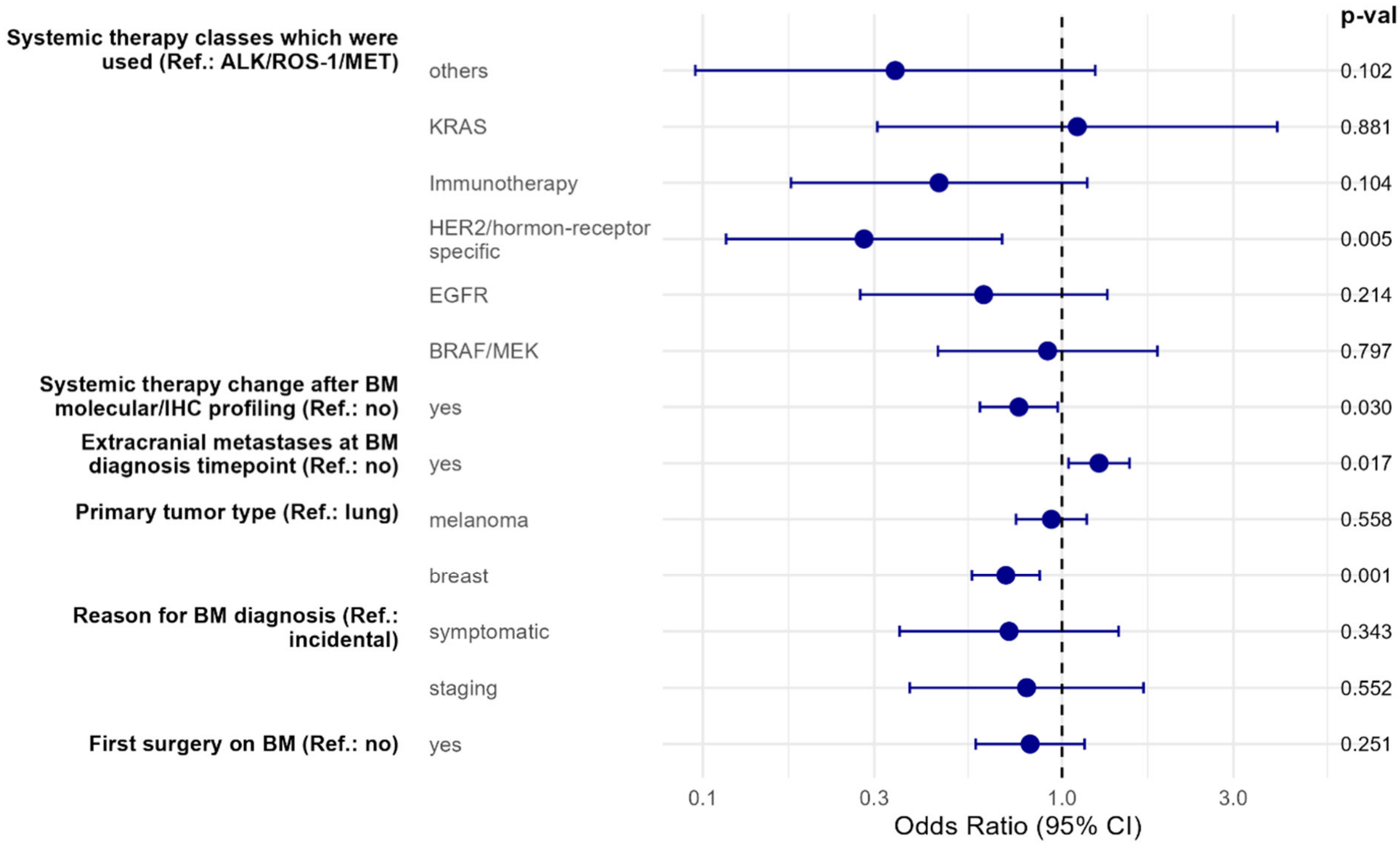

Supplementary Figure 3: Cox proportional hazards model of OS

Cox Proportional-Hazards model on OS for selected parameters (in bold) with the respective reference values in brackets.

BM brain metastases; OS overall survival; IHC immunohistochemistry; CI confidence interval
